# LINC-PINT suppresses cisplatin resistance in gastric cancer by inhibiting autophagy activation *via* epigenetic silencing of ATG5 by EZH2

**DOI:** 10.3389/fphar.2022.968223

**Published:** 2022-08-25

**Authors:** Cheng Zhang, Tong Kang, Xinyi Wang, Jizhao Wang, Lin Liu, Jiawei Zhang, Xu Liu, Rong Li, Jiansheng Wang, Jia Zhang

**Affiliations:** ^1^ Department of Thoracic Surgery, The First Affiliated Hospital of Xi’an Jiaotong University, Xi’an, Shaanxi, China; ^2^ Department of Dermatology, The Second Affiliated Hospital of Xi’an Jiaotong University, Xi’an, Shaanxi, China; ^3^ Department of Radiotherapy, The First Affiliated Hospital of Xi’an Jiaotong University, Xi’an, Shaanxi, China

**Keywords:** LINC-PINT, autophagy, DDP-resistance, gastric cancer, Atg5

## Abstract

Resistance to cisplatin (DDP) is a major obstacle in the clinical treatment of advanced gastric cancer (GC). Long noncoding RNA (lncRNA) play a significant regulatory role in the development and drug resistance of GC. In this study, we reported that the lncRNA LINC-PINT was downregulated in DDP-resistant GC cells. Functional studies showed that LINC-PINT inhibited proliferation and migration of DDP-resistant GC cells *in vitro*, and overexpression of LINC-PINT could enhance the sensitivity of DDP-resistant GC cells to DDP. Further investigation revealed that LINC-PINT recruited enhancer of zeste homolog 2 (EZH2) to the promotor of ATG5 to inhibit its transcription, leading to the suppression of autophagy and DDP resensitization. Collectively, our results revealed how the LINC-PINT/EZH2/ATG5 axis regulates autophagy and DDP resistance in GC. These data suggest that LINC-PINT may be a potential therapeutic target in GC.

## Introduction

Gastric cancer (GC) is one of the most common malignancies and the third leading cause of cancer-related deaths worldwide, especially in Asia (Bray et al., 2018). There were 1,033,701 newly diagnosed GC cases and 782,685 deaths caused by GC worldwide in 2018 (Bray et al., 2018). Surgical resection is considered the preferred option for early GC patients, while perioperative and postoperative chemotherapy is important to prevent relapse and metastasis (Smyth et al., 2020). Drug resistance remains a handicap for cancer chemotherapy eligibility (Orditura et al., 2014). The mechanisms underlying drug resistance of GC are still unknown. Therefore, exploring the mechanisms underlying GC chemoresistance is valuable for identifying novel treatments.

Autophagy is an important self-protection mechanism of cells that occurs in destructive environments, such as nutrient deficiency, drug toxicity, and hypoxia (Singletary and Milner, 2008; Ouyang et al., 2012). Autophagy establishes drug resistance in cancer cells by recovering energy, eliminating damaged organelles, and degrading harmful elements (Ouyang et al., 2012). Autophagy is regulated by several factors, including autophagy-related genes, microRNAs, TP53, and PI3K-AKT-MTOR signaling pathways (Kumar et al., 2012; Wu et al., 2014). Accumulating research has provided evidence that autophagy participates in cisplatin (DDP) resistance in variety of cancers (Song et al., 2009; He et al., 2015). Furthermore, inhibition of autophagy can sensitize GC cells to DDP (Dong et al., 2016; Zhao et al., 2016). Although a few studies have revealed the regulatory mechanism underlying chemoresistance and autophagy in GC, further study is needed.

Long non-coding RNAs (lncRNAs) are a family of RNAs that lack protein-coding potential and are characterized by a length of more than 200 nucleotides (Quinn and Chang, 2016). Numerous studies have indicated that lncRNAs participate in many important cancer pathological processes, such as cancer initiation, development, and metastasis (Cech and Steitz, 2014; Schmitt and Chang, 2016; Jiang et al., 2019). Recently, increasing evidence has indicated that lncRNAs play important roles in drug resistance (Wei et al., 2019). The main mechanisms of lncRNAs-induced drug resistance, including effects on the cell cycle, apoptosis, cancer stem cells, epithelial-mesenchymal transition, and autophagy, have been reported in many recent studies (Wei et al., 2019). These studies indicate lncRNAs are a potential target for GC chemoresistance.

Recent studies have reported that the lncRNA, p53-induced transcript (LINC-PINT) is downregulated in several cancer types, including colon cancer, lung cancer, breast cancer, glioblastoma, and GC (Marin-Bejar et al., 2013; Marin-Bejar et al., 2017; He et al., 2021; Bukhari et al., 2022). LINC-PINT directly interacts with the polycomb repressive complex 2 (PRC2) to regulate the expression of downstream genes (Marin-Bejar et al., 2013; Marin-Bejar et al., 2017). PRC2 catalyzes the tri-methylation of histone H3 at lysine 27 (H3K27me3), which is a mark of silent chromatin (Bracken and Helin, 2009; Margueron and Reinberg, 2011; Sauvageau et al., 2013). However, the function of LINC-PINT in GC is still undefined.

In this study, we describe the down-regulation of lncRNA LINC-PINT in DDP-resistant GC cells. We confirm that LINC-PINT promotes the increased sensitivity of GC cells to DDP by suppressing autophagy. LINC-PINT recruits the enhancer of zeste homolog 2 (EZH2) to the promotor of ATG5, leading to the increased the level of H3K27me3 and epigenetic silencing.

## Materials and methods

### Culture and establishment of DDP-resistant cell lines

Human GC cell lines AGS, MGC-803, HGC-27 and human normal gastric epithelial cell line GES-1 were obtained from Cell Bank, Chinese Academy of Sciences. AGS cells were cultured in F12-K medium (Gibco, CA, USA). Other cells were cultured in RPMI 1640 medium (Gibco, CA, USA). All media were supplemented with 10% fetal bovine serum (Gibco, CA, USA), 1% penicillin and streptomycin. All cells were maintained in a 37°C incubator with 5% CO_2_.

DDP-resistant cell lines (AGS-DDP and MGC-803-DDP) were established by exposure to increasing concentration of DDP. Non-resistant AGS and MGC-803 cells were cultured with 2 μg/ml DDP for 24 h, then the DDP-free medium was replaced, and cell viability was observed. The above process was repeated until AGS, and MGC-803 cells overcame DDP inhibition. Then the concentration of DDP was increased to 3, 4, 6, 8, 10 μg/ml. DDP resistance was detected by assessing the IC50.

### Patients

A total of 156 paired GC tissues and paracancerous tissues were collected from patients with GC, who were pathologically diagnosed from 2014 January to 2015 December at the First Affiliated hospital of Xi’an Jiaotong University. All the patients were informed of the study and provided signed consent to participate. This study was approved by the Ethics Committee of First Affiliated hospital of Xi’an Jiaotong University and was performed according to the Declaration of Helsinki.

### Quantitative real-time PCR analysis

Total RNA was extracted using RNAiso Plus (TaKaRa Biotechnology, Dalian, China). RNA was converted to cDNA by PrimeScript™ RT reagent Kit (TaKaRa Biotechnology, Dalian, China). Quantitative real-time PCR assays were performed using SYBR Premix Ex TaqTM II (TaKaRa Biotechnology, Dalian, China). β-actin and U6 were used as the internal control for expression quantitation and the 2-ΔΔCt method was used for analysis. Primer sequences are listed in Supplementary Table S1.

### Vectors and cell transfection

Lentivirus vectors of LINC-PINT overexpression and negative controls were obtained from Genechem Biotech (Shanghai, China). Ubi-MCS-SV40-Cherry-IRES-puromycin was used as the vector for LINC-PINT overexpression and negative controls. Lentivirus were transduced into cells following the manufacturer’s instructions. Stable transfected cell lines were selected with 5 μg/ml puromycin. The LINC-PINT overexpression plasmid was synthesized by Genechem Biotech (Shanghai, China). GV219 was used as an expression vector for the LINC-PINT overexpression plasmid. siRNA and the negative control were obtained from GenePharma (Shanghai, China).

Cell transfection was performed using the Lipofectamine 3,000 transfection reagent (Thermo Fisher Scientific, Shanghai, China) following manufacturer’s instructions. The siRNA sequences are available in Supplementary Table S2.

### Protein extraction and western blotting

Total protein was extracted using RIPA buffer (Beyotime), supplemented with protease inhibitor. Protein concentrations were validated by the BCA assay (Sigma–Aldrich, Cambridge, MA, USA). Proteins were separated by SDS–PAGE gels and were then transferred to polyvinylidene difluoride membranes (Millipore). The membranes were blocked with 5% fat-free milk in TBS-T for 2 h at room temperature. Next, primary antibodies were incubated overnight at 4°C. The following day, secondary antibodies were incubated for 1.5 h at room temperature. The primary antibodies were anti-LC3 (14600-1-AP, Proteintech, USA), anti-p62 (ab207305, Abcam, USA), anti-EZH2 (#5246, Cell Signaling Technology, USA), anti-ATG5 (10181-2-AP, Proteintech, USA), and anti-GAPDH (10494-1-AP, Proteintech, USA).

### Colony formation assay

GC cells were seeded in six-well plates at a density of 500 cells per well with 10 μg/ml DDP for 48 h. Subsequently, cells were cultured with fresh medium with 10% FBS for 2 weeks. Then, 4% paraformaldehyde were used for 15 min. Colonies were stained with 0.1% aqueous crystal violet for 15 min.

### Cell counting kit-8 assay

To detect the drug resistance of all the cell lines, the Cell Counting Kit-8 Assay (CCK-8, Dojindo, Kumamoto, Japan) was used to evaluate the viability of cells treated with different concentrations of DDP. Cells were seeded into 96-well plates (800 cells per well) and cultured with 0, 2, 4, 6, 8, 10, 12, 14 μg/ml DDP. After culturing for 24 h, fresh complete medium with 10% CCK-8 was replaced and then cells were incubated for an additional 2 h. The absorbance at 450 nm was measured. The IC50 was calculated using GraphPad Prism 8.2 (GraphPad Software, La Jolla, CA, USA).

### ChIP assay

The ChIP assay was performed using the EZ-Magna ChIP™ A/G Chromatin Immunoprecipitation Kit (Sigma–Aldrich, Darmstadt, Germany). Cells were treated with 1% formaldehyde to co-precipitate DNA with proteins at room temperature for 10 min. Then cells were lysed using cell Lysis Buffer with 1% Protease Inhibitor Cocktail II. The cell lysate was sonicated to obtain 200–1,000 bp DNA fragments. Sheared chromatin was immunoprecipitated using anti-EZH2 (#5246, Cell Signaling Technology, USA) and anti-H3K27me3 (#9733, Cell Signaling Technology, USA) antibodies using protein A/G magnetic beads. IgG antibody was used as a negative control. The ChIPed DNA was purified and analyzed by qPCR.

### RNA pull-down assay

The RNA pull-down assay was performed as described previously (Marin-Bejar and Huarte, 2015). Briefly, Biotinylated LINC-PINT and negative control were synthesized using T7 RNA Polymerase (Cat. No. 10881767001, Roche) and Biotin RNA Labeling Mix (Cat. No. 11685597910, Roche). The Biotinylated RNA was incubated with protein lysate. After incubation, the coprecipitated proteins were retrieved by streptavidin beads. After elution of the magnetic beads, western blotting was performed to detect the RNA-associating proteins.

### Transwell assay

Cells (30,000 per chamber) were seeded into the upper chamber of Transwell plates with FBS-free medium for migration assays. The upper chambers with Matrigel-coated membranes were used for invasion tests. The complete medium supplemented with 10% FBS was supplied to the lower chambers. The cells were then cultured at 37°C and 5% CO_2_ for 24 h. All the medium was removed and the cells on the upper chamber were gently wiped off. The cells were stained with 0.1% aqueous crystal violet for 15 min and then counted under an optical microscope.

### Autophagic flux assay

For autophagic flux analysis, cells were transfected with the mRFP-GFP-LC3 plasmid (GenePharma, Shanghai, China). Cells were then seeded into glass dishes for 72 h. The accumulation and distribution of mRFP-GFP-LC3 or mRFP-LC3 puncta were observed and captured by a Leica laser scanning confocal microscope (Leica, TCS SP5, Japan).

### Xenograft tumor model

All animal experiments were performed in accordance with national and international guidelines. Four-week-old female nude mice were purchased from the Animal center of Xi’an Jiaotong University and housed in pathogen-free conditions with free access to food and water. All mice were divided into four groups (six mice per group). AGS, AGS-DDP, AGS-DDP LINC-PINT overexpression, and relevant NC cells were injected subcutaneously into the left flank of mice (5×10^6^ cells per mouse). DDP (3 mg/kg) was intraperitoneally administered 1 week after cell injection and was given twice weekly for 2 weeks. Tumor volumes were calculated by the equation: (length × width^2^) × 0.5. After 4 weeks, xenograft tumors were collected for further analysis.

### Statistical analysis

All statistical analysis was conducted using SPSS 20.0 statistical software (IBM, Armonk, NY, USA) and GraphPad Prism 8.0 (GraphPad Software, La Jolla, CA, USA). Data are presented as mean ± standard deviation (SD). ImageJ Pro was used to quantify the western blotting results. Student’s t test, χ^2^ test, or the Wilcoxon test, as appropriate was used to determine the significance of differences between groups. A *p*-value < 0.05 was considered to indicate statistical significance.

## Results

### LINC-PINT was downregulated in human GC tissues and was associated with DDP resistance

**FIGURE 1:**
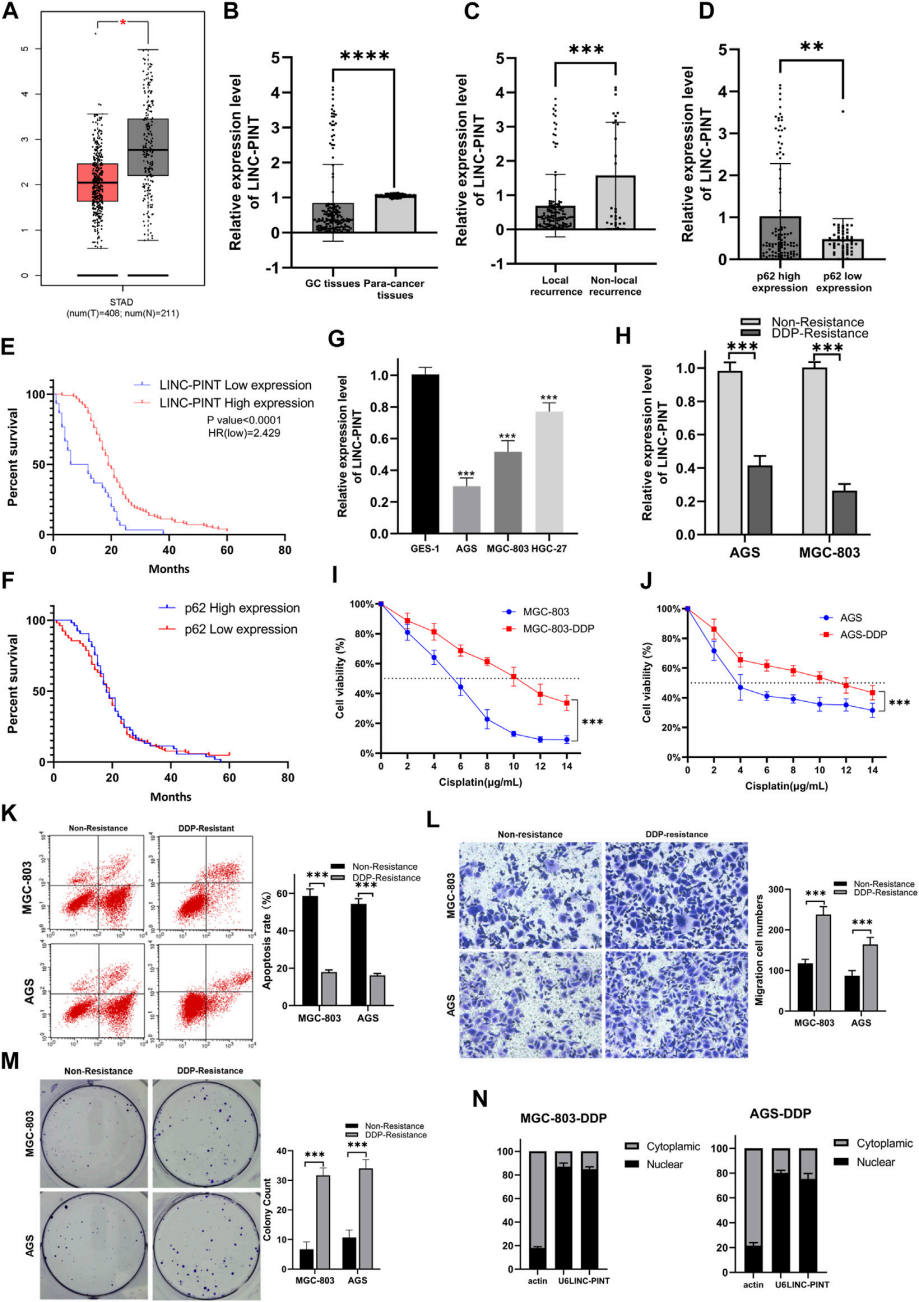
**(A)** The expression of LINC-PINT in gastric cancer tissues versus adjacent normal tissues based on the GEPIA database (Gene Expression Profiling Interactive Analysis database; gepia.cancer-pku.cn). **(B)** The expression of LINC-PINT in 156 paired gastric cancer tissues and paracancerous tissues. **(C)** The expression of LINC-PINT in local recurrence gastric cancer tissues and non-local recurrence paracancerous tissues. **(D)** LINC-PINT expression in gastric cancer tissues with high expression of p62 and low expression of p62. **(E)** Kaplan–Meier analysis for overall survival of patients with low and high expression of LINC-PINT. **(F)** Kaplan–Meier analysis for overall survival of patients with low and high expression of p62. **(G)** LINC-PINT expression in GC cell lines and normal gastric cell lines. **(H)** LINC-PINT expression in DDP-resistant GC cell lines and non-resistant cell lines. **(I)** CCK-8 assay in MGC-803 and MGC-803-DDP evaluating the IC50 of DDP. **(J)** CCK-8 assay for AGS and AGS-DDP evaluating the IC50 of DDP. **(K)** Flow cytometry evaluating the apoptosis of DDP-resistant and non-resistant GC cells. **(L)** Transwell assays evaluating the migration ability of DDP-resistant and non-resistant GC cells. **(M)** Colony formation assay of DDP-resistant and non-resistant GC cells. **(N)** Subcellular localization of LINC-PINT in AGS-DDP and MGC-803-DDP cell lines. Data are represented as the mean ± SD. *p < 0.05. **p < 0.005. ***p < 0.001. The experiments were repeated independently at least three times.

The Cancer Genome Atlas (TCGA) was used to extract data regarding the expression of LINC-PINT, which was found to be significantly downregulated in cancer tissues compared with normal tissues (Figure 1A). We then analyzed the expression of LINC-PINT in 156 paired samples of GC tissues and paracancerous tissues by quantitative real-time PCR (qRT-PCR). The results showed that LINC-PINT was significantly downregulated in GC tissues compared with paracancerous tissues (Figure 1B). Moreover, LINC-PINT had a lower expression in patients presenting local recurrence of GC than in GC patients with non-local recurrence (Figure 1C). Local recurrence indicated resistance toward chemotherapy. Given the relationship between autophagy and chemoresistance, we investigated the correlation between LINC-PINT and p62 expression. LINC-PINT levels were lower in patients that have low expression of p62 (Figure 1D). The lower expression of p62 indicated more active autophagy processes. Kaplan–Meier analysis revealed that patients with lower LINC-PINT expression had shorter overall survival (OS) while p62 expression had no significant influence on survival (Figures 1E,F).

The expression of LINC-PINT was measured in GC cell lines. The qRT-PCR showed that the expression of LINC-PINT was downregulated in GC cell lines AGS, HGC-27, MGC-803, compared with the normal gastric epithelial cell line GES-1 (Figure 1G). Moreover, we established DDP-resistant AGS (AGS-DDP) and MGC-803 (MGC-803-DDP) cell lines. The resistance of AGS-DDP and MGC-803-DDP was confirmed by measuring the half-maximal inhibitory concentration (IC50) of AGS-DDP and MGC-803-DDP compared with the non-resistant cells after treating with DDP (Figures 1I,J). We found LINC-PINT was decreased in DDP-resistant GC cells when compared with the DDP-sensitive cells (Figure 1H). Flow cytometry showed that DDP-resistant cells had a significantly lower percentages of apoptotic cells compared with non-resistant cells after treated with 5 μg/ml DDP (Figure 1K). Wound healing assay and transwell assay indicated that DDP-resistant cells had a stronger migration ability (Figures 1L,M). Furthermore, the qRT-PCR results demonstrated that LINC-PINT was mostly located in the nuclear fraction (Figure 1N).

### LINC-PINT was involved in DDP-resistance in GC

**FIGURE 2:**
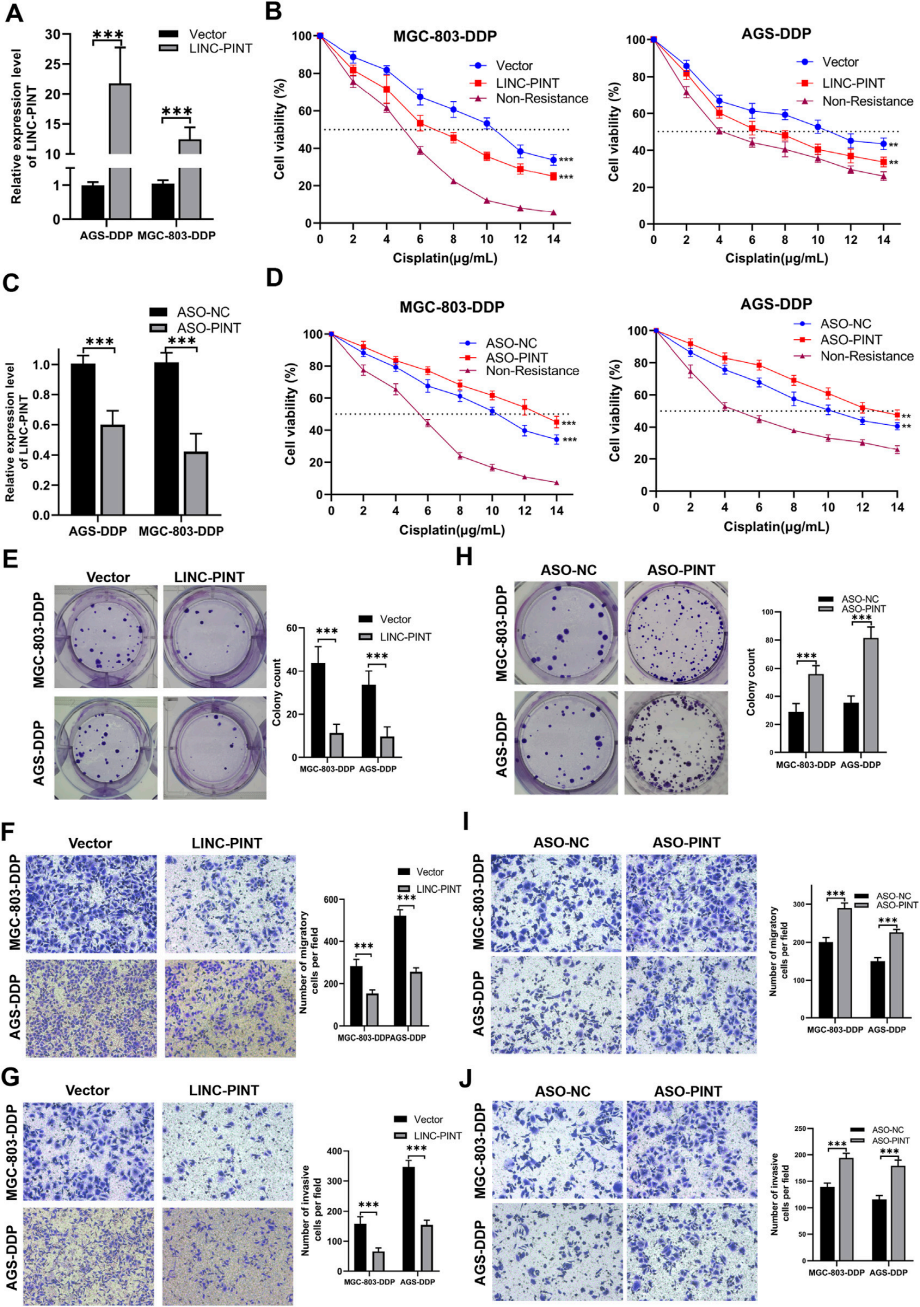
LINC-PINT involvement in DDP-resistance in GC. **(A)** LINC-PINT expression in LINC-PINT-overexpressing AGS-DDP and MGC-803-DDP cells. **(B)** CCK-8 assays in LINC-P INT-overexpressing AGS-DDP and MGC-803-DDP cells. **(C)** LINC-PINT expression in LINC-PINT-silenced AGS-DDP and MGC-803-DDP cells. **(D)** CCK-8 assays in LINC-PINT-silenced AGS-DDP and MGC-803-DDP cells. **(E)** Colony formation assay of LINC-PINT-overexpressing AGS-DDP and MGC-803-DDP cells. **(F)** Transwell assays evaluating the migration ability of LINC-PINT-overexpressing AGS-DDP and MGC-803-DDP cells. **(G)** Transwell assays evaluating the invasion ability of LINC-PINT-overexpressing AGS-DDP and MGC-803-DDP cells. **(H)** Colony formation assay of LINC-PINT-silenced AGS-DDP and MGC-803-DDP cells. **(I)** Transwell assays evaluating the migration ability of LINC-PINT-silenced AGS-DDP and MGC-803-DDP cells. **(J)** Transwell assays evaluating the invasion ability of LINC-PINT-silenced AGS-DDP and MGC-803-DDP cells. Data represent the mean ± SD. *p < 0.05. **p < 0.005. ***p < 0.001. The experiments were repeated independently at least three times.

To explore the biological activity of LINC-PINT in GC cells, we overexpressed or silenced LINC-PINT expression in DDP-resistant GC cells (AGS-DDP and MGC-803-DDP) (Figures 2A,C). Next, the CCK-8 and colony formation assays were carried out to evaluate DDP-sensitivity. The results showed LINC-PINT overexpression could significantly enhance the DDP-sensitivity with 10 μg/ml DDP (Figures 2B,E), while silencing LINC-PINT displayed the opposite results (Figures 2D,H). These data indicated the potential relationship between LINC-PINT and DDP-resistance. Furthermore, the migration and invasion ability of LINC-PINT-overexpressing DDP-resistant GC cells was significantly decreased whereas silencing LINC-PINT increased the capacity for migration and invasion (Figures 2F,G,I,J, Supplementary Figures S1A,B). The apoptotic rate of LINC-PINT-overexpressing DDP-resistant GC cells was increased (Supplementary Figures S1C). In contrast, silencing LINC-PINT decreased the apoptotic rate (Supplementary Figures S1D).

### Autophagy was induced by DDP and contributed to chemoresistance in GC

**FIGURE 3:**
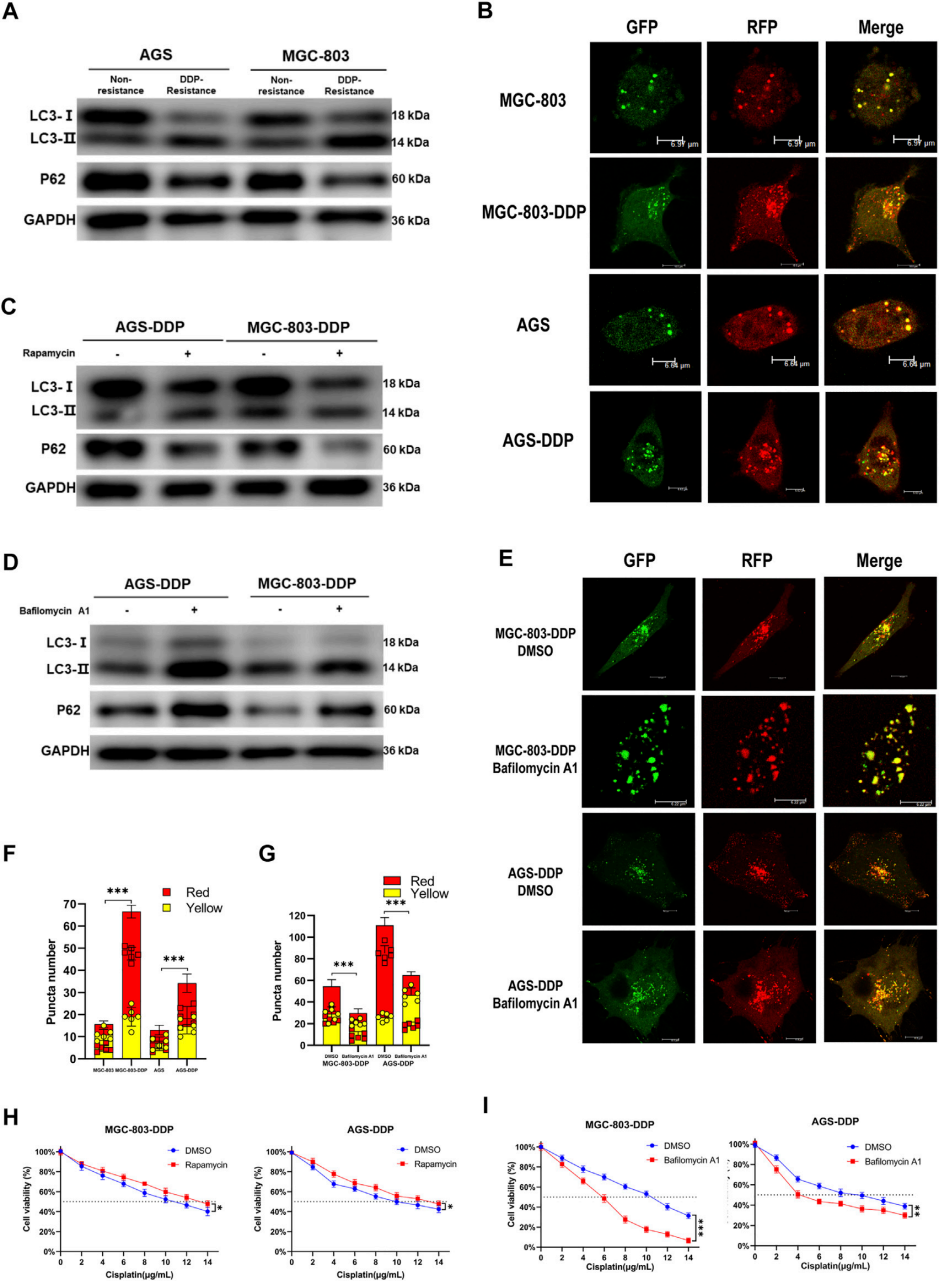
Autophagy contributes to DDP-resistance in GC. **(A)** Western blotting of autophagy-related protein LC3 and p62 in AGS-DDP, MGC-803-DDP, and relative non-resistant cells. **(B)** Confocal microscopy showing the autophagy flux in AGS-DDP and MGC-803-DDP cells. **(C)** The effects of rapamycin on LC3-II and p62 levels. **(D)** The effects of bafilomycin A1 on LC3 and p62 levels. **(E)** The effects of bafilomycin A1 on autophagy flux in AGS-DDP and MGC-803-DDP cells. **(F)** Statistical analysis of red and yellow puncta number in AGS-DDP and MGC-803-DDP cells compared to non-resistant cells [cell image: **(B)**]. **(G)** Statistics of red and yellow puncta number in AGS-DDP and MGC-803-DDP cells treated with bafilomycin A1 [cell image: **(E)**]. **(H)** The effects of bafilomycin A1 on the IC50 of AGS-DDP and MGC-803-DDP cells. **(I)** The effects of rapamycin on the IC50 of AGS-DDP and MGC-803-DDP cells. Data are represented as the mean ± SD. *p < 0.05. **p < 0.005. ***p < 0.001. The experiments were repeated independently at least three times.

The promotional effect of autophagy on chemoresistance of cancer has been reported in many studies. Based on our findings above indicating that LINC-PINT regulated the chemoresistance of GC cells, we hypothesized that LINC-PINT might regulate DDP-resistance by modulating autophagy. Thus, we evaluated the influence of DDP on GC cell autophagy. We observed that LC3-II was highly expressed, whereas p62 had a lower expression in DDP-resistant cells, compared with the non-resistant cells (Figure 3A). Confocal microscopy confirmed enhanced autophagy flux in DDP-resistant cells (Figures 3B,F). To further explore the relationship between autophagy and DDP-resistance in GC, rapamycin or bafilomycin A1 were used to enhance or inhibit autophagy, respectively, in DDP-resistant cells and non-resistant cells. As shown in Figure 3C, rapamycin, an autophagy enhancer, significantly increased the expression of LC3-II along with decreased p62 expression in non-resistant cells. Next, bafilomycin A1, an autophagy inhibitor, was applied to DDP-resistant cells. Western blotting showed that bafilomycin A1 increased the levels of LC3-II (Figure 3D), which has been validated previously (Yamamoto et al., 1998; Jahreiss et al., 2008; Klionsky et al., 2008). Bafilomycin A1 blocks autophagosome-lysosome fusion, which led to an accumulation of LC3-II, to disrupts autophagic flux. The autophagic flux assay confirmed the inhibition of autophagy (Figures 3E,G). After the confirmation of autophagy levels, the CCK-8 assay was used to evaluate sensitivity to DDP. Bafilomycin A1 significantly decreased the IC50 of AGS-DDP and MGC-803-DDP compared with DMSO (Figure 3H). In contrast, rapamycin could increase the IC50 of DDP sensitive cells (Figure 3I). Besides, we detected the expression of LINC-PINT after treated with rapamycin or bafilomycin A1. Rapamycin treatment decreased LINC-PINT expression whereas bafilomycin A1 increased LINC-PINT expression (Supplementary Figures S2A,B). Therefore, we assumed that the activation of autophagy could promote DDP resistance.

### LINC-PINT inhibited DDP-resistance by attenuating autophagy

**FIGURE 4:**
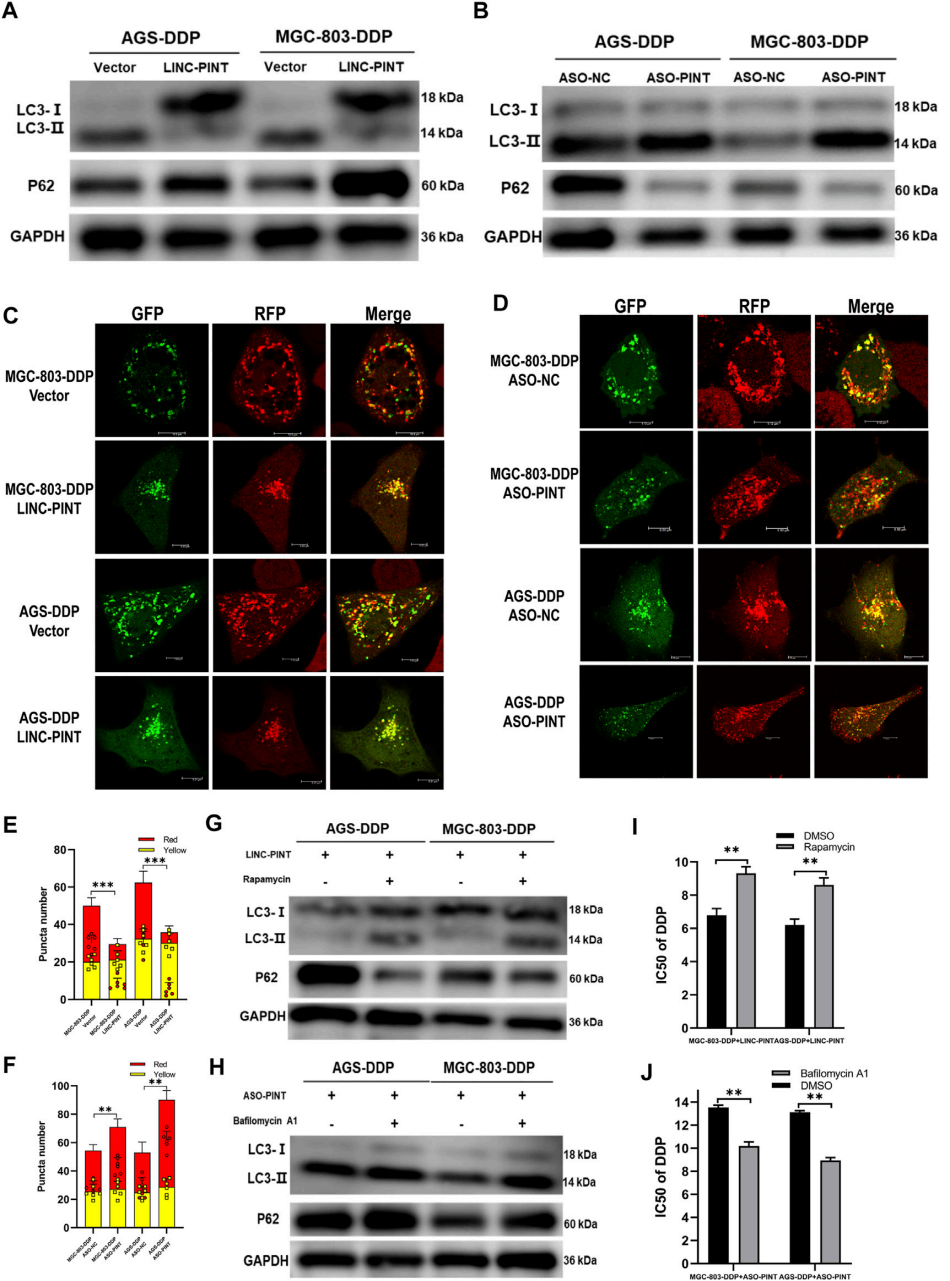
LINC-PINT inhibited DDP-resistance via attenuated autophagy. **(A)** Western blotting for LC3 and p62 in LINC-PINT overexpressed AGS-DDP andMGC-803-DDP cells. **(B)** Western blotting for LC3 and p62 in LINC-PINT silenced AGS-DDP and MGC-803-DDP cells. **(C)** Confocal microscopy to observe autophagy flux in LINC-PINT overexpressed MGC-803-DDP and AGS-DDP cells. **(D)** Confocal microscopy to observe autophagy flux in LINC-PINT silenced MGC-803-DDP and AGS-DDP cells. **(E)** Statistics of red and yellow puncta number in LINC-PINT overexpressed MGC-803-DDP and AGS-DDP cells [cell image: **(C)**]. **(F)** Statistics of red and yellow puncta number in LINC-PINT silenced AGS-DDP and MGC-803-DDP cells [cell image: **(D)**]. **(G)** Western blotting for LC3 and p62 in LINC-PINT overexpressed MGC-803-DDP and AGS-DDP cells treated with Rapamycin. **(H)** Western blotting for LC3 and p62 in LINC-PINT silenced MGC-803-DDP and AGS-DDP cells treated with Bafilomycin A1. **(I)** CCK-8 assay for LINC-PINT overexpressed MGC-803-DDP and AGS-DDP cells treated with Rapamycin to evaluate the IC50 of DDP. **(J)** CCK-8 assay for LINC-PINT silenced MGC-803-DDP and AGS-DDP cells treated with Bafilomycin A1 to evaluate the IC50 of DDP. Data were represented as the mean ± SD. *p < 0.05. **p < 0.005. ***p < 0.001. The experiments were repeated independently at least three times.

Next, we investigated whether LINC-PINT could regulate autophagy in GC cells. Following LINC-PINT overexpression in DDP-resistant cells, LC3B-II expression was inhibited and p62 expression was increased (Figure 4A). Likewise, confocal microscopy revealed that the numbers of autophagosomes significantly decreased in LINC-PINT overexpressed cells (Figures 4C,E). Silencing LINC-PINT led to the opposite effects (Figures 4B,D,F). These results indicate that LINC-PINT regulated cell autophagy.

To confirm whether LINC-PINT regulates DDP-resistance via regulation of autophagy, we exposed GC cells to the autophagy-regulating drugs, rapamycin and bafilomycin A1, to alter autophagy. Rapamycin reversed the inhibition of LINC-PINT overexpression on DDP-resistance (Figures 4G,I), while Bafilomycin A1 weakened the acceleration of LINC-PINT silencing on DDP-resistance (Figures 4H,J). Taken together, these results indicate that LINC-PINT inhibited DDP-resistance in GC cells by attenuating cell autophagy.

### ATG5 is a target of LINC-PINT

**FIGURE 5:**
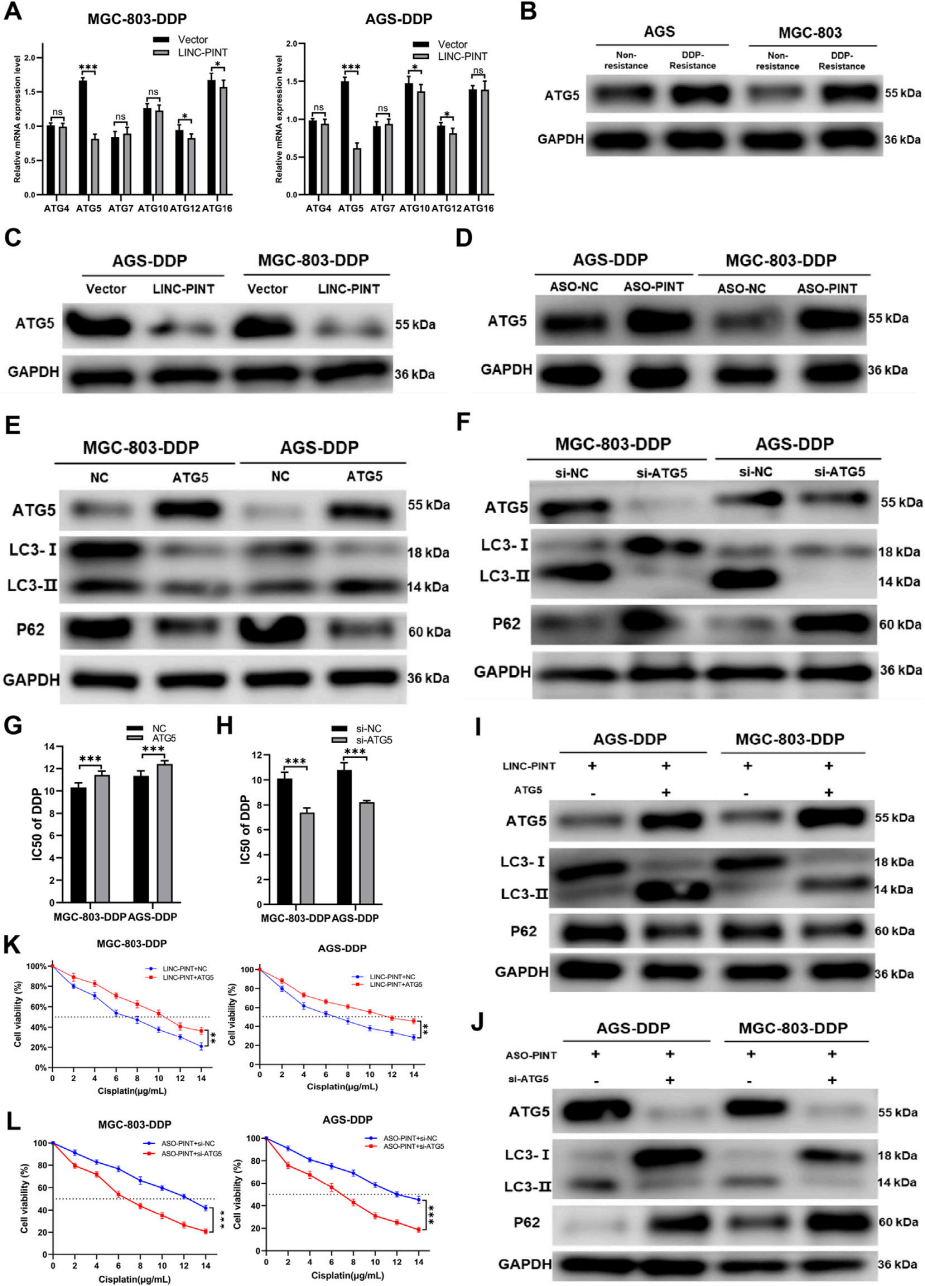
ATG5 is a target of LINC-PINT. **(A)** qRT-PCR analysis ATGs expression in MGC-803-DDP and AGS-DDP cells with LINC-PINT overexpression or not. **(B)** Western blotting for ATG5 expression in DDP-resistant GC cells. **(C)** Western blotting for ATG5 expression in LINC-PINT overexpressed AGS-DDP cells and MGC-803-DDP cells. **(D)** Western blotting for ATG5 expression in LINC-PINT silenced AGS-DDP cells and MGC-803-DDP cells. **(E)** Western blotting for ATG5, LC3 and p62 in ATG5 overexpressed MGC-803-DDP and AGS-DDP cells. **(F)** Western blotting for ATG5, LC3 and p62 in ATG5 silenced MGC-803-DDP and AGS-DDP cells. **(G)** CCK-8 assay for ATG5 overexpressed MGC-803-DDP and AGS-DDP cells to evaluate the IC50 of DDP. **(H)** CCK-8 assay for ATG5 silenced MGC-803-DDP and AGS-DDP cells to evaluate the IC50 of DDP. **(I)** Western blotting for ATG5, LC3 and p62 in LINC-PINT and ATG5 overexpressed MGC-803-DDP and AGS-DDP cells. **(J)** Western blotting for ATG5, LC3 and p62 in LINC-PINT and ATG5 silenced MGC-803-DDP and AGS-DDP cells. **(K)** CCK-8 assay for LINC-PINT and ATG5 overexpressed MGC-803-DDP and AGS-DDP cells to evaluate the IC50 of DDP. **(L)** CCK-8 assay for LINC-PINT and ATG5 silenced MGC-803-DDP and AGS-DDP cells to evaluate the IC50 of DDP.

To investigate the molecular mechanism through which LINC-PINT regulates autophagy, we performed RNA transcriptome sequencing (RNA-seq) to identify the differently expressed genes and transcripts in LINC-PINT overexpressed GC cells and control cells. The results showed that the expression of autophagy-related genes (ATGs) differed significantly (Supplementary Figure S3). Thus, we measure the expression of ATGs by qRT-PCR (Figure 5A) and found that ATG5 was the most significantly decreased gene in LINC-PINT overexpressing cells. Western blotting indicated that the expression of ATG5 was enhanced in DDP-resistant cells (Figure 5B). Furthermore, LINC-PINT overexpression significantly reduced ATG5 protein levels, while LINC-PINT silencing showed the opposite effects (Figures 5C,D).

ATG5 plays a key role in autophagosome formation. The ATG5-ATG12-ATG6L1 complex acts as an E3-like enzyme to catalyze the conversion from LC3B-I to LC3B-II (Romanov et al., 2012). To explore the function of ATG5 in autophagy and DDP-resistance, ATG5 was overexpressed or silenced in MGC-803-DDP and AGS-DDP cells, respectively and the overexpression and silencing efficiency of ATG5 was verified by western blotting (Figures 5E,F). Subsequently, the effects of ATG5 on autophagy were assessed. Overexpression of ATG5 decreased the level of autophagy while silencing of ATG5 triggered the opposite results (Figures 5E,F). The CCK-8 assay indicated ATG5 overexpression enhanced the DDP-resistance of MGC-803-DDP and AGS-DDP cells while ATG5 silencing weakened the DDP-resistance (Figures 5G,H).

To investigate whether LINC-PINT could influence the autophagy and DDP-resistance of MGC-803-DDP and AGS-DDP cells by regulating ATG5, we overexpressed ATG5 in LINC-PINT overexpressed MGC-803-DDP and AGS-DDP cells simultaneously. As displayed in Figure 5I, ATG5 overexpression reversed LINC-PINT-mediated autophagy inhibition. The CCK-8 assay verified ATG5 could increase DDP-resistance in LINC-PINT-overexpressing MGC-803-DDP and AGS-DDP cells (Figure 5K). Next, we silenced ATG5 in LINC-PINT depleted cells. Western blotting and CCK-8 assays revealed the opposite results (Figures 5J,L). Taken together, LINC-PINT suppressed the resistance of GC cells to DDP by regulating ATG5.

### LINC-PINT regulates ATG5 *via* EZH2

**FIGURE 6:**
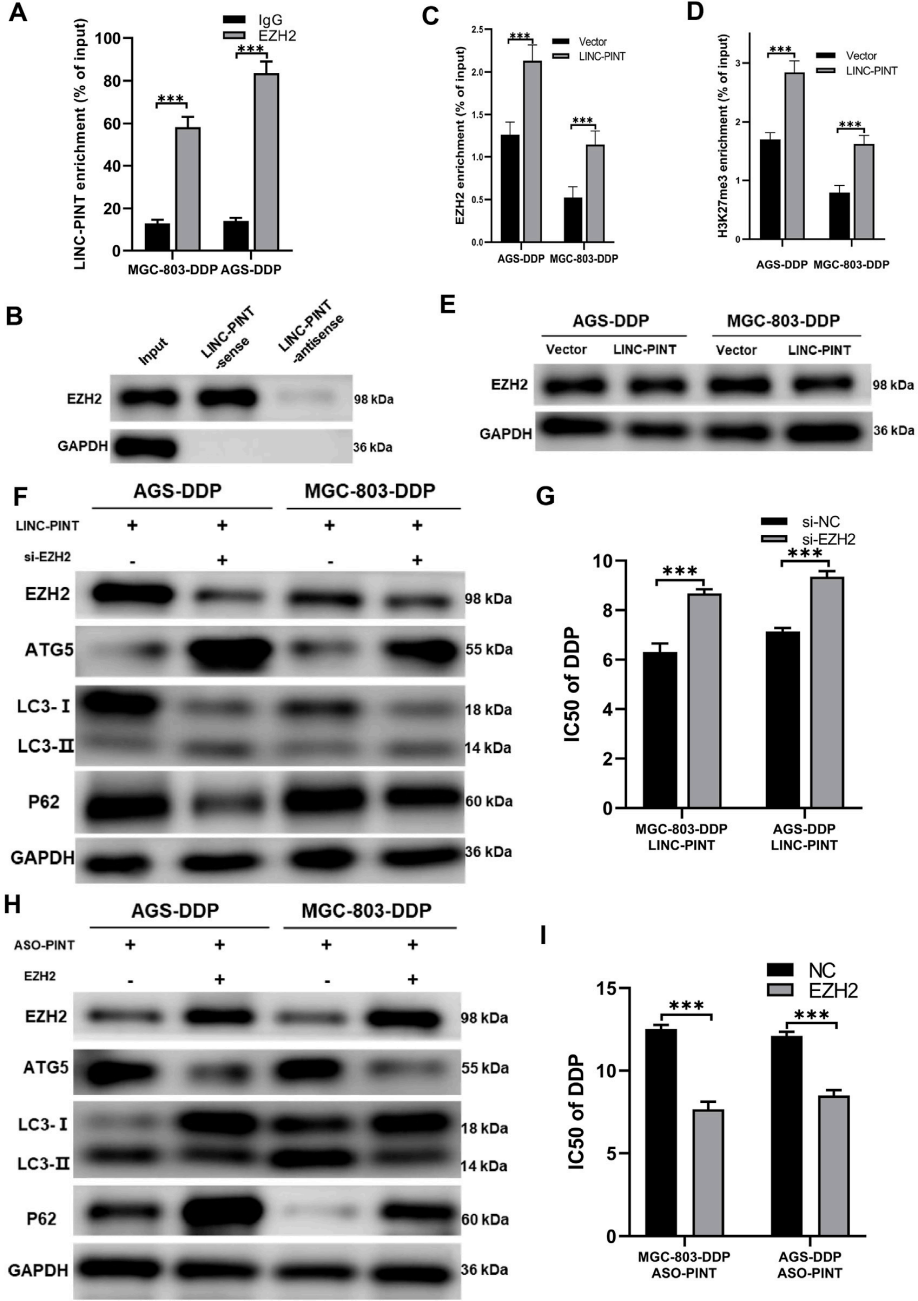
LINC-PINT regulates ATG5 via EZH2. **(A)** RIP assay showing the interaction between LINC-PINT and EZH2. **(B)** RNA pull-down assay showing the interaction between LINC-PINT and EZH2. **(C)** ChIP assay showing EZH2 enrichment in the promoter region of ATG5 of LINC-PINT-overexpressing DDP-resistant cells. **(D)** ChIP assay showing H3K27me3 enrichment in the promoter region of ATG5 in controls or LINC-PINT-overexpressing DDP-resistant cells. **(E)** Western blotting showing EZH2 expression in LINC-PINT-overexpressed DDP-resistant cells. **(F)** Western blotting showing EZH2, ATG5, LC3 and p62 expression in LINCPINT-overexpressed and EZH2 silenced DDP-resistant cells. **(G)** CCK-8 assay for LINC-PINT-overexpressed and EZH2-silenced DDP-resistant cells to evaluate the IC50 of DDP. **(H)** Western blotting evaluating EZH2, ATG5, LC3, and p62 expression in LINC-PINT-silenced and EZH2 overexpressed DDP-resistant cells. **(I)** CCK-8 assay for LINC-PINT-silenced and EZH2-overexpressed DDP-resistant cells to evaluate the IC50 of DDP. Data are represented as the mean ± SD. *p < 0.05. **p < 0.005. ***p < 0.001. The experiments were repeated independently at least three times.

Previous study showed that LINC-PINT interacts with PRC2 to regulate gene expression (Marin-Bejar et al., 2017). As shown in Figure 1F, LINC-PINT showed nuclear localization. We then performed an RNA Immunoprecipitation (RIP) assay and RNA pull-down assay to confirm the interaction between LINC-PINT and EZH2 in MGC-803 cells (Figures 6A,B). We then investigated whether LINC-PINT the expression of ATG5 by EZH2. The chromatin immunoprecipitation (ChIP) assay in GC cells revealed that binding of EZH2 to the promoter of ATG5 was significantly increased following LINC-PINT overexpression (Figure 6C). Concomitant with the increased EZH2 occupancy, the levels of H3K27me3, an epigenetic modification catalyzed by EZH2, was significantly increased (Figure 6D). Furthermore, LINC-PINT overexpression did not influence the expression of EZH2 (Figure 6E). These results indicated that LINC-PINT could recruit EZH2 to the promotor of ATG5.

To verify whether LINC-PINT regulated DDP-resistance of GC cells via epigenetic modification by EZH2 on ATG5, we simultaneously overexpressed LINC-PINT and silenced EZH2 in MGC-803-DDP and AGS-DDP cells. Silencing EZH2 reversed the inhibition of LINC-PINT on ATG5 and autophagy (Figure 6F). IC50 of MGC-803-DDP and AGS-DDP increased after EZH2 silencing which was consistent with the increased levels of autophagy (Figure 6G). Overexpression of EZH2 in LINC-PINT-silenced cells showed a similar relationship (Figures 6H,I). In conclusion, these results suggested that LINC-PINT silenced the transcription of ATG5 by recruiting EZH2 in GC cells.

### LINC-PINT overexpression inhibited DDP resistance *in vivo*

**FIGURE 7:**
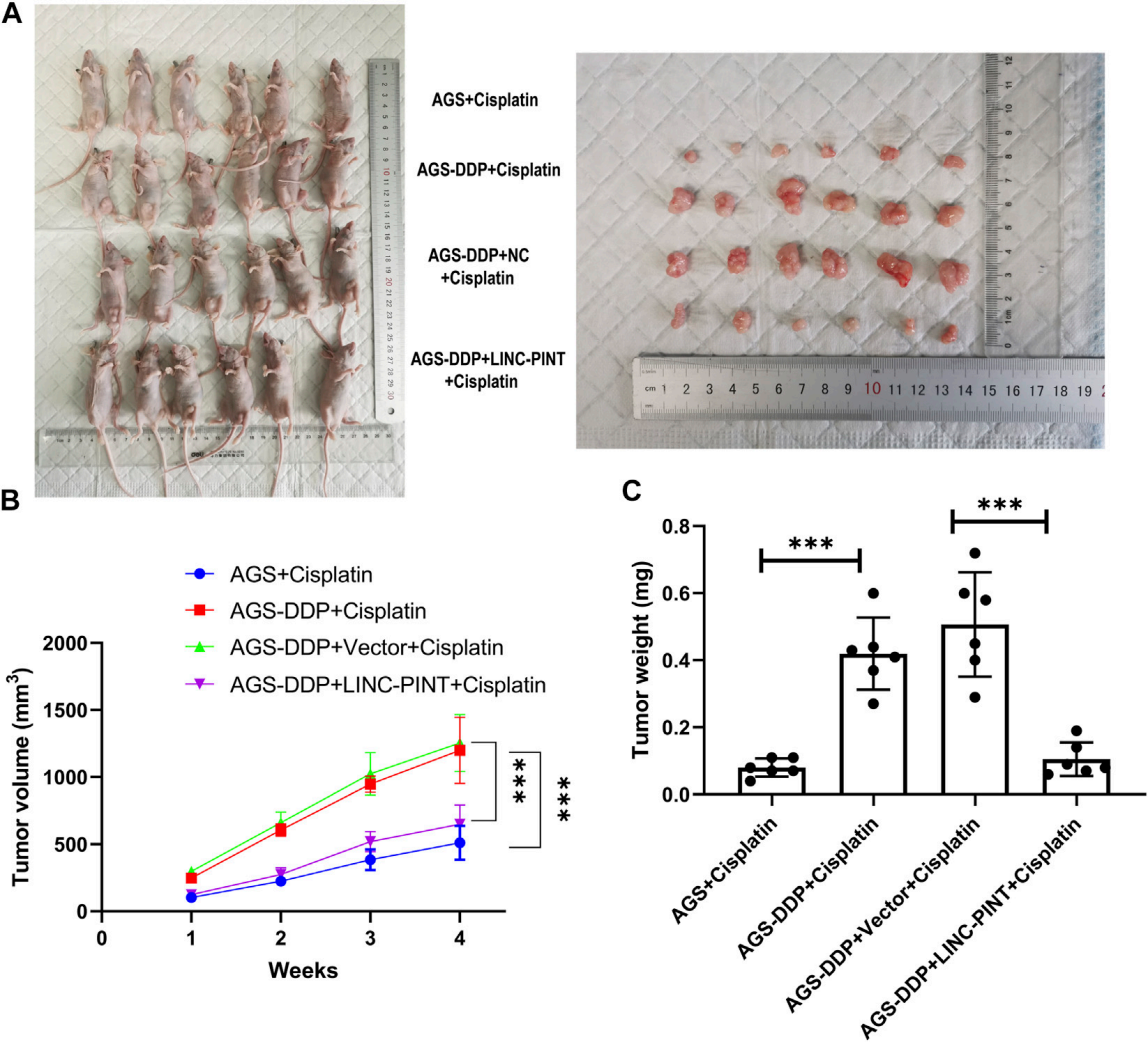
Effect of LINC-PINT on tumor development *in vivo*. **(A)** The nude mouse model of LINC-PINT-overexpressed DDP-resistant cells and control DDP-resistant cells. **(B)** Average volume of transplanted tumors from respective groups. Tumor volume was measured every 3 days. **(C)** Average weight of transplanted tumors from respective groups. Data were represented as the mean ± SD. *p < 0.05. **p < 0.005. ***p < 0.001.

We investigated the effects of LINC-PINT on DDP-resistance in tumor-bearing nude mice treated with DDP. As displayed in Figures 7A–C, tumors weights and volumes with LINC-PINT overexpression were significantly smaller than those in the control group. In addition, the sensitivity to DDP of tumor harboring LINC-PINT overexpression was significantly increased. Overall, these data suggest that LINC-PINT could promote the sensitivity of GC cells to DDP *in vivo*.

## Discussion

Chemotherapy resistance continues to be the main obstacle in tumor treatment (Ali et al., 2012). Further investigations aimed at understanding the mechanisms underlying chemoresistance are urgently needed. DDP is one of the most common drugs used in GC chemotherapy regimens (Bahar et al., 2019). Multiple mechanisms regulate DDP resistance, such as epigenetic regulation, defective DNA repair, and enhanced drug clearance (Helleday et al., 2008; Xiang et al., 2014). Growing evidence has indicated that autophagy plays an important role in chemotherapy resistance and promotion of survival (Desantis et al., 2018). In this study, we defined a novel model the downregulation of lncRNA LINC-PINT enhanced autophagy by targeting EZH2-induced epigenetic silencing ATG5, which promoted DDP resistance in GC cells.

LncRNAs participate in many pathological processes of cancer, such as proliferation, migration, invasion, and metastasis (Dhamija and Diederichs, 2016). In GC, many abnormally expressed lncRNAs have been recognized to regulate cancer progression (Zhang et al., 2019), targeted therapy sensitivity (Liu et al., 2014; Wei et al., 2018), and chemotherapy resistance (Xi et al., 2019). Studies have shown that LINC-PINT plays a key role in tumor suppressor activity and is downregulated in many cancers (Marin-Bejar et al., 2017). Jia et al. (2020) reported that the downregulation of LINC-PINT is associated with cancer cell growth and invasion. LINC-PINT inhibited DNA damage repair via the ATM/ATR-Chk1/Chk2 signaling pathways (Wang et al., 2021). Furthermore, LINC-PINT could interact with DNA-PKcs to increase radiosensitivity. However, there has been no research concentrating on the involvement of LINC-PINT in chemoresistance in GC. In this study, we found that LINC-PINT was downregulated in GC patients, and patients with low LINC-PINT had the shorter overall survival. Besides, the patients with local recurrence had the lower LINC-PINT expression compared with non-local recurrence patients. Local recurrence indicated the chemoresistance in GC patients. Based on the above findings, we constructed DDP-resistant AGS and MGC-803 cells. The results of qRT-PCR assay showed that LINC-PINT was significantly downregulated in AGS-DDP and MGC-803-DDP cells compared to non-resistant cells. Besides, overexpression of LINC-PINT could resensitize DDP-resistant GC cells to DPP treatment, whereas silencing LINC-PINT enhanced the DDP-resistance in GC cells. These results indicate the roles of LINC-PINT in DDP-resistance of GC.

Recently, autophagy has been proven to be an important mechanism involved in inducing chemoresistance (Lefort et al., 2014; Corazzari et al., 2015). Our study showed that autophagy was activated in AGS-DDP and MGC-803-DDP cells and the activation of autophagy contributed to chemoresistance. As previously reported, lncRNAs can regulate autophagy in many cancer types. Xin et al. (2019) found that lncRNA HULC promoted autophagy and enhanced DDP resistance in GC cells. Our group previously reported that lncRNA NORAD could enhance autophagy flux by the miR-433–3p/ATG5-ATG12 axis leading to oxaliplatin resistance (Wang et al., 2021). In this study, we proved that LINC-PINT overexpression decreased the ratio of LC3 II/LC3 I and inhibited the autophagy flux, and LINC-PINT silencing enhanced autophagy conversely. Our findings indicated that LINC-PINT decreased DDP-resistance by suppressing autophagy in DDP-resistant GC cells. Of course, further study is needed to better understand the mechanisms underlying LINC-PINT in the management of autophagy and DDP-resistance in DDP-resistant GC cells.

Previous studies have indicated that lncRNAs might act as epigenetic modifiers to control gene expression in different cancers (Gupta et al., 2010; Yap et al., 2010; Tseng et al., 2014). Marin-Bejar et al. (2013), Marin-Bejar et al. (2017) reported that LINC-PINT directly interacts with PRC2 to regulate the expression of downstream genes. EZH2 is the catalytic subunit of PRC2. Epigenetically, PRC2-mediated gene silence is dependent on the level of EZH2-mediated H3K27me3 (Schuettengruber and Cavalli, 2009; Simon and Kingston, 2009). Growing evidence shows that lncRNAs participate in EZH2-mediated tumor regulation. LncRNA HOTAIR recruits EZH2 to target genes to induce the trimethylation of H3K27 and achieve the epigenetic silencing of metastasis-suppressor genes (Gupta et al., 2010). LncRNA ANCR interacts with EZH2 to promote its phosphorylation which facilitates EZH2 degradation (Li et al., 2017). In this study, we confirmed that LINC-PINT could interact directly with EZH2 using RIP and RNA pull-down assays. The RNA-seq of LINC-PINT-overexpressing DDP-resistant GC cells indicated that ATG5 might be a downstream gene of LINC-PINT. Combined with ChIP and western blotting analyses, we confirmed that LINC-PINT silenced the expression of ATG5 by recruiting EZH2 to the promotor region of ATG5 and generated H3K27me3. Moreover, our results showed that ATG5 silencing by LINC-PINT was EZH2-dependent. ATG5 exerts a key role in autophagy (Mizushima et al., 1998). The ATG5-ATG12/ATG16 complex is localized to the cell membrane and if any individual components of this complex are deleted, this will result in the failure of autophagosome formation (Romanov et al., 2012; Walczak and Martens, 2013). Our study was the first to clarify that lncRNA LINC-PINT regulates the expression of ATG5 via EZH2-dependent epigenetic mechanism.

Although this study revealed a new mechanism of DDP-resistance in GC, there are still some limitations needed to be considered. First, only two DDP-resistant GC cell lines were used in this study. More DDP-resistant GC cell lines will be more convincing. Second, this study more focused on the experiments *in vitro*. Further animal experiments could improve the quality of this study.

In conclusion, our study provided evidence supporting the role of LINC-PINT in DDP-resistant GC cells. Additional, experiments revealed that the LINC-PINT/EZH2/ATG5 pathway was involved in DDP-resistance of GC, which suggested that targeting LINC-PINT may be a potential therapeutic strategy for reversing DDP resistance.

## Data Availability

The original contributions presented in the study are publicly available. This data can be found here: [PRJNA 860835].
