# Corrigendum: LINC-PINT suppresses cisplatin resistance in gastric cancer by inhibiting autophagy activation *via* epigenetic silencing of ATG5 by EZH2

**DOI:** 10.3389/fphar.2025.1424320

**Published:** 2025-03-14

**Authors:** Cheng Zhang, Tong Kang, Xinyi Wang, Jizhao Wang, Lin Liu, Jiawei Zhang, Xu Liu, Rong Li, Jiansheng Wang, Jia Zhang

**Affiliations:** ^1^ Department of Thoracic Surgery, The First Affiliated Hospital of Xi’an Jiaotong University, Xi’an, Shaanxi, China; ^2^ Department of Dermatology, The Second Affiliated Hospital of Xi’an Jiaotong University, Xi’an, Shaanxi, China; ^3^ Department of Radiotherapy, The First Affiliated Hospital of Xi’an Jiaotong University, Xi’an, Shaanxi, China

**Keywords:** LINC-PINT, autophagy, DDP-resistance, gastric cancer, Atg5

In the published article, there was an error in Figures 2, 5, 6 as published. In Figure 2H, the wrong picture was used for AGS-DDP ASO-PINT. The error does not affect the conclusions of the experiment. In Figure 5D, we mistakenly duplicated and uploaded Figure 6B into Figure 5D. In Figures 5, 6, Figures 5J, 6F were reversed due to lack of proficiency with the software**.** The corrected Figures 2, 5, 6 and their captions appear below.

**FIGURE 2:**
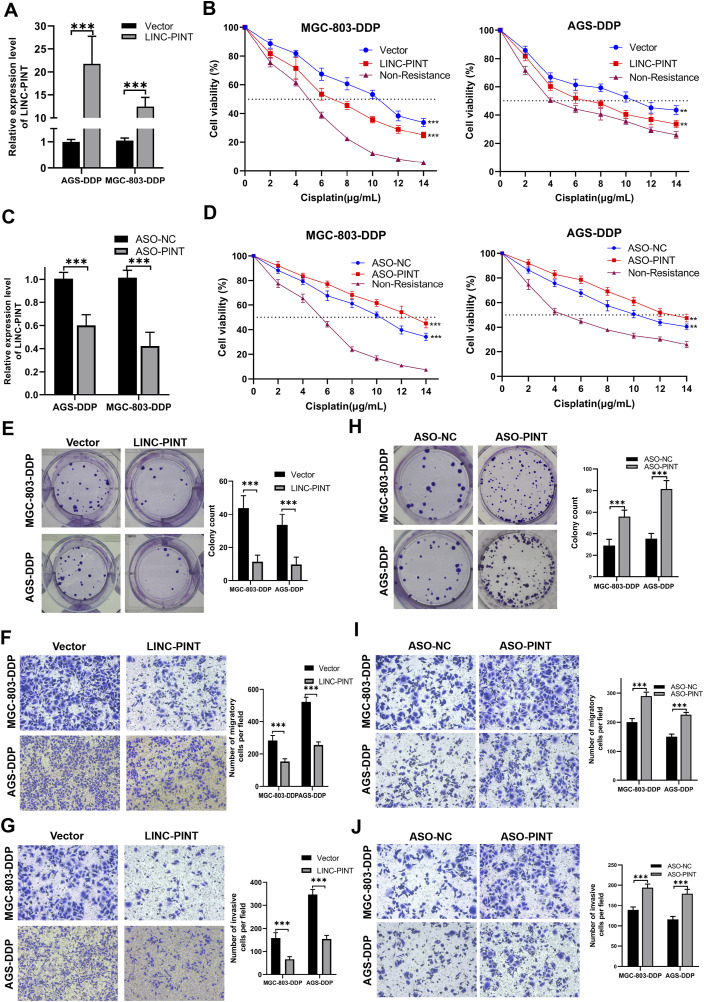
LINC-PINT involvement in DDP-resistance in GC. **(A)** LINC-PINT expression in LINC-PINT-overexpressing AGS-DDP and MGC-803-DDP cells. **(B)** CCK-8 assays in LINC-P INT-overexpressing AGS-DDP and MGC-803-DDP cells. **(C)** LINC-PINT expression in LINC-PINT-silenced AGS-DDP and MGC-803-DDP cells. **(D)** CCK-8 assays in LINC-PINT-silenced AGS-DDP and MGC-803-DDP cells. **(E)** Colony formation assay of LINC-PINT-overexpressing AGS-DDP and MGC-803-DDP cells. **(F)** Transwell assays evaluating the migration ability of LINC-PINT-overexpressing AGS-DDP and MGC-803-DDP cells. **(G)** Transwell assays evaluating the invasion ability of LINC-PINT-overexpressing AGS-DDP and MGC-803-DDP cells. **(H)** Colony formation assay of LINC-PINT-silenced AGS-DDP and MGC-803-DDP cells. **(I)** Transwell assays evaluating the migration ability of LINC-PINT-silenced AGS-DDP and MGC-803-DDP cells. **(J)** Transwell assays evaluating the invasion ability of LINC-PINT-silenced AGS-DDP and MGC-803-DDP cells. Data represent the mean ± SD. *p < 0.05. **p < 0.005. ***p < 0.001. The experiments were repeated independently at least three times.

**FIGURE 5:**
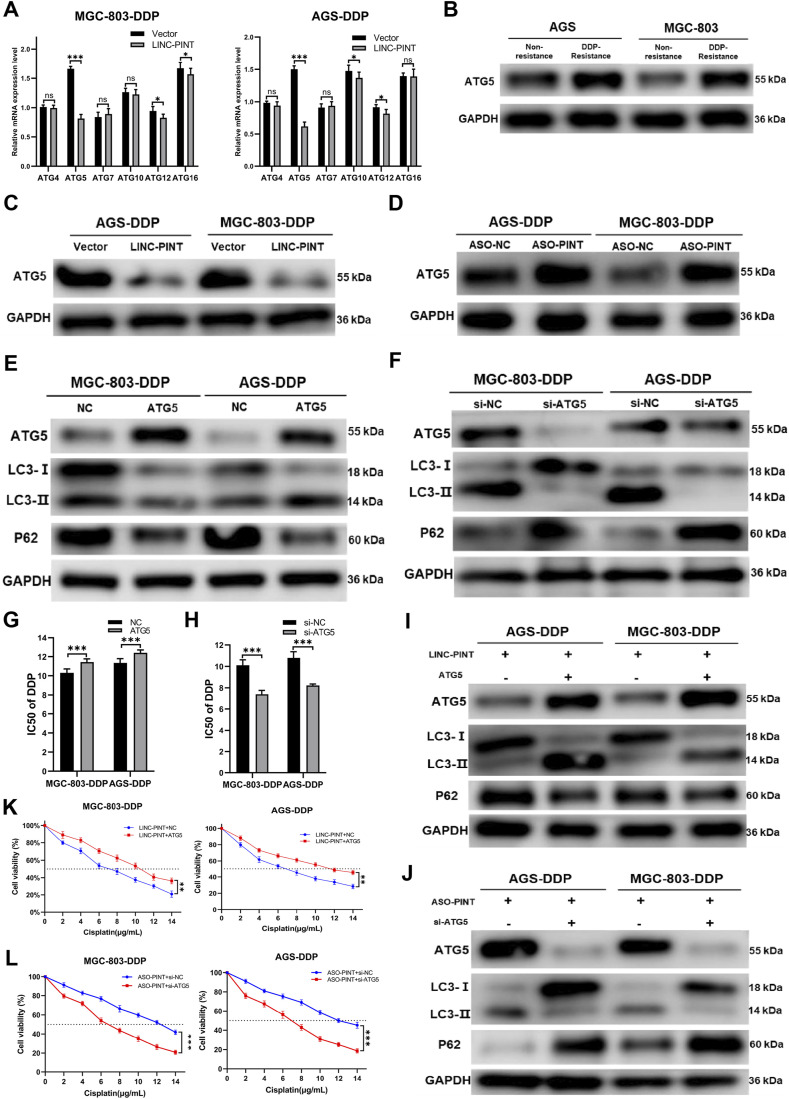
ATG5 is a target of LINC-PINT. **(A)** qRT-PCR analysis ATGs expression in MGC-803-DDP and AGS-DDP cells with LINC-PINT overexpression or not. **(B)** Western blotting for ATG5 expression in DDP-resistant GC cells. **(C)** Western blotting for ATG5 expression in LINC-PINT overexpressed AGS-DDP cells and MGC-803-DDP cells. **(D)** Western blotting for ATG5 expression in LINC-PINT silenced AGS-DDP cells and MGC-803-DDP cells. **(E)** Western blotting for ATG5, LC3 and p62 in ATG5 overexpressed MGC-803-DDP and AGS-DDP cells. **(F)** Western blotting for ATG5, LC3 and p62 in ATG5 silenced MGC-803-DDP and AGS-DDP cells. **(G)** CCK-8 assay for ATG5 overexpressed MGC-803-DDP and AGS-DDP cells to evaluate the IC50 of DDP. **(H)** CCK-8 assay for ATG5 silenced MGC-803-DDP and AGS-DDP cells to evaluate the IC50 of DDP. **(I)** Western blotting for ATG5, LC3 and p62 in LINC-PINT and ATG5 overexpressed MGC-803-DDP and AGS-DDP cells. **(J)** Western blotting for ATG5, LC3 and p62 in LINC-PINT and ATG5 silenced MGC-803-DDP and AGS-DDP cells. **(K)** CCK-8 assay for LINC-PINT and ATG5 overexpressed MGC-803-DDP and AGS-DDP cells to evaluate the IC50 of DDP. **(L)** CCK-8 assay for LINC-PINT and ATG5 silenced MGC-803-DDP and AGS-DDP cells to evaluate the IC50 of DDP. Data were represented as the mean ± SD. *p < 0.05. **p < 0.005. ***p < 0.001. The experiments were repeated independently at least three times.

**FIGURE 6:**
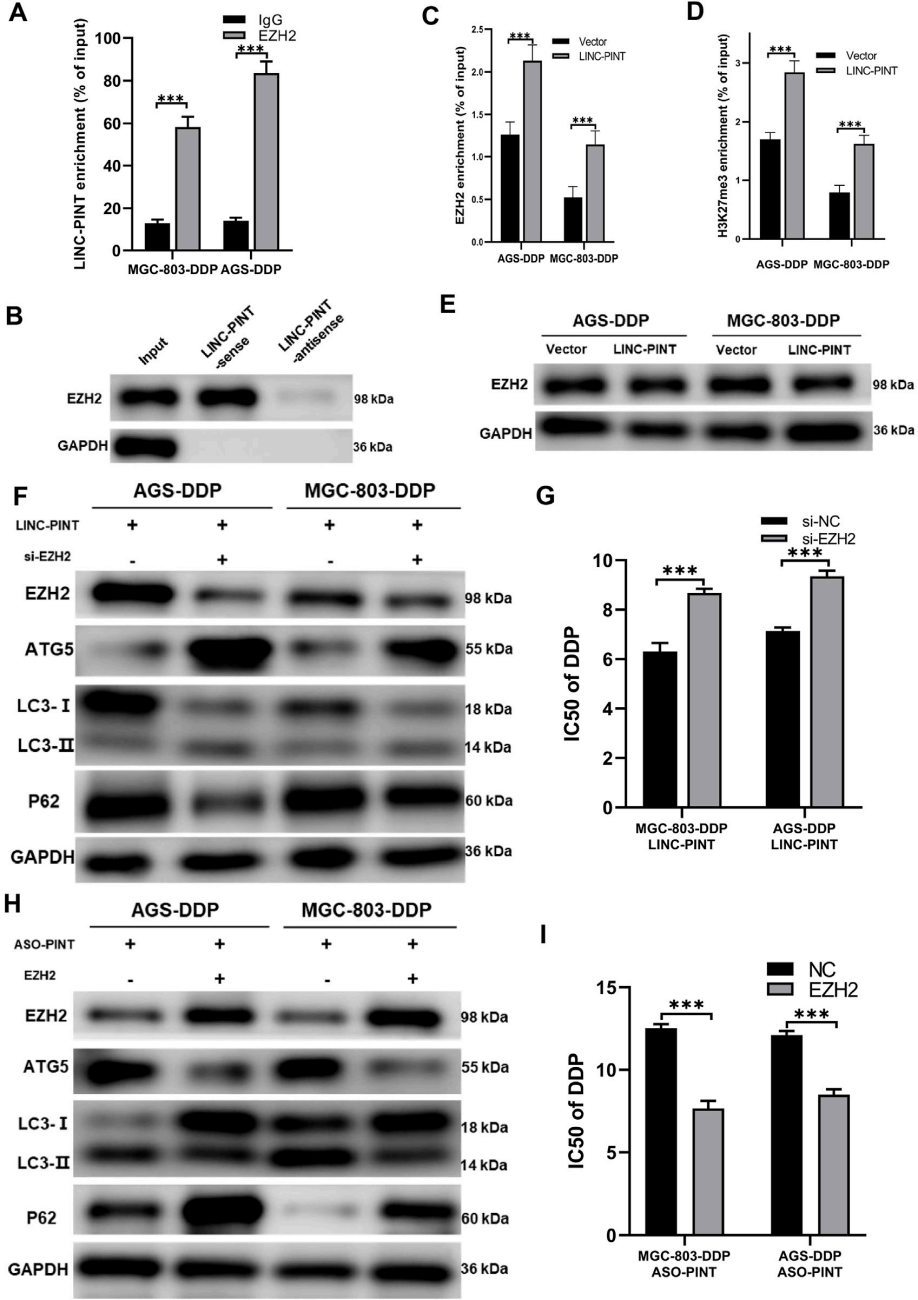
LINC-PINT regulates ATG5 via EZH2. **(A)** RIP assay showing the interaction between LINC-PINT and EZH2. **(B)** RNA pull-down assay showing the interaction between LINC-PINT and EZH2. **(C)** ChIP assay showing EZH2 enrichment in the promoter region of ATG5 of LINC-PINT-overexpressing DDP-resistant cells. **(D)** ChIP assay showing H3K27me3 enrichment in the promoter region of ATG5 in controls or LINC-PINT-overexpressing DDP-resistant cells. **(E)** Western blotting showing EZH2 expression in LINC-PINT-overexpressed DDP-resistant cells. **(F)** Western blotting showing EZH2, ATG5, LC3 and p62 expression in LINC-PINT-overexpressed and EZH2 silenced DDP-resistant cells. **(G)** CCK-8 assay for LINC-PINT-overexpressed and EZH2-silenced DDP-resistant cells to evaluate the IC50 of DDP. **(H)** Western blotting evaluating EZH2, ATG5, LC3, and p62 expression in LINC-PINT-silenced and EZH2 overexpressed DDP-resistant cells. **(I)** CCK-8 assay for LINC-PINT-silenced and EZH2-overexpressed DDP-resistant cells to evaluate the IC50 of DDP. Data are represented as the mean ± SD. *p < 0.05. **p < 0.005. ***p < 0.001. The experiments were repeated independently at least three times.

In the published article, there was an error in **Supplementary Data Sheet 2**. Data Sheet 2 is the original western blots. The article was revised after peer review to include some additional images. When uploading the final version of the original western blots, we mistakenly uploaded the unmodified version. The error does not affect the conclusions of the experiment. The correct **Supplementary Data Sheet 2** has been published with the original article.

The authors apologize for these errors and state that this does not change the scientific conclusions of the article in any way. The original article has been updated.

