# Supplementary material for: LINC-PINT suppresses cisplatin resistance in gastric cancer by inhibiting autophagy activation via epigenetic silencing of ATG5 by EZH2
Source: Front Pharmacol. 2022 Aug 25;13:968223. doi: 10.3389/fphar.2022.968223 (PMC9452659; doi:10.3389/fphar.2022.968223)

Figure 3A

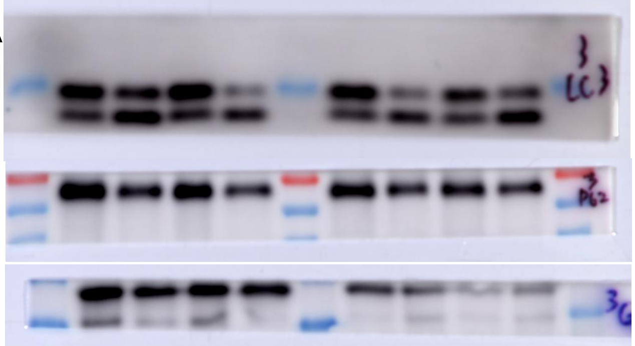

Figure 3C

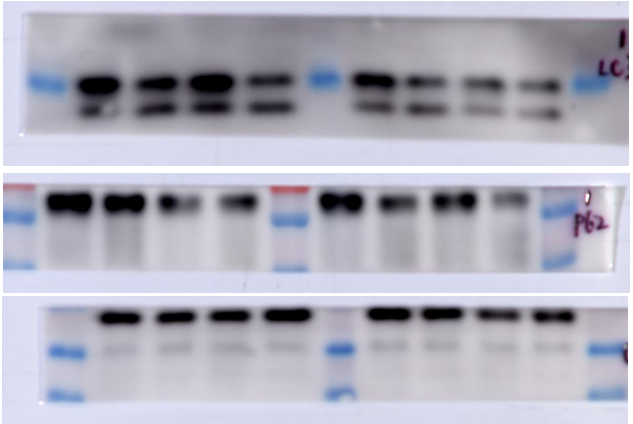

Figure 3D

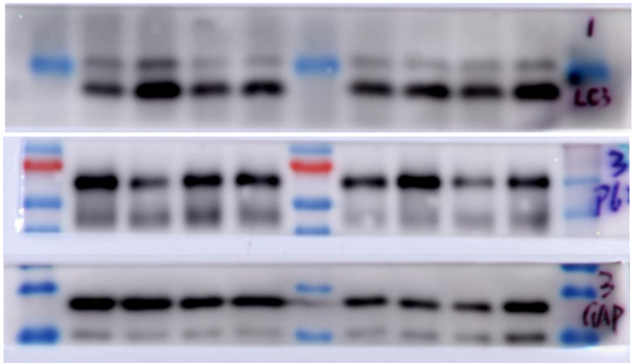

Figure 4A

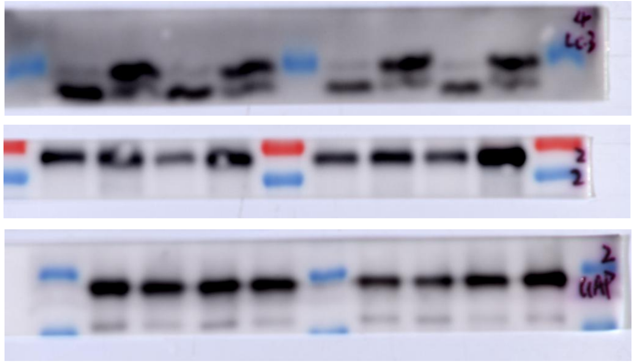

Figure 4B

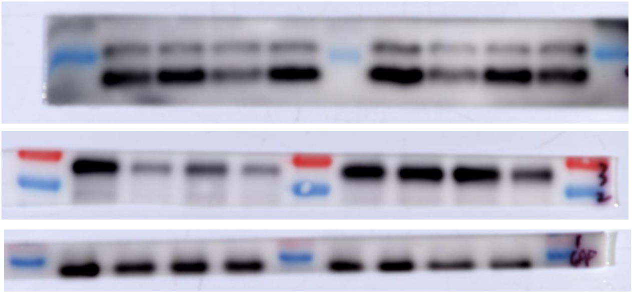

Figure 4G

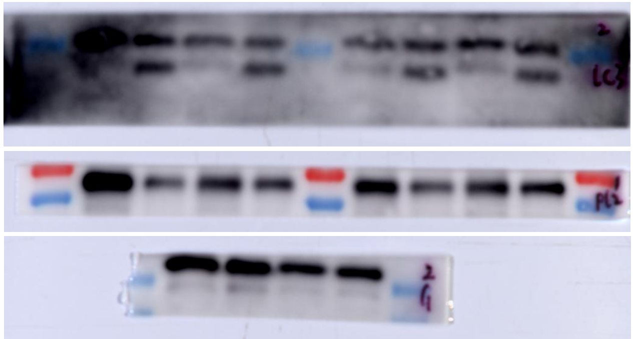

Figure 4H

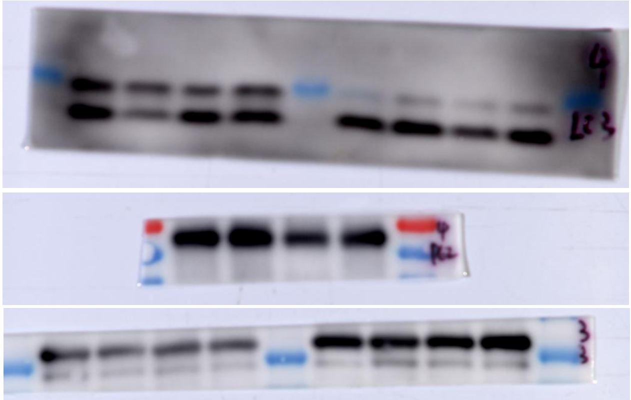

Figure 5B

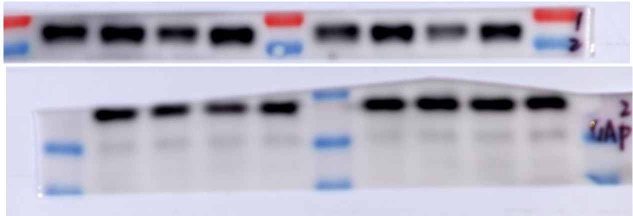

Figure 5C

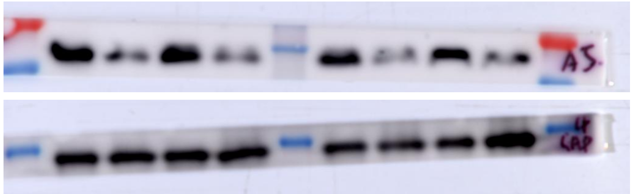

Figure 5D

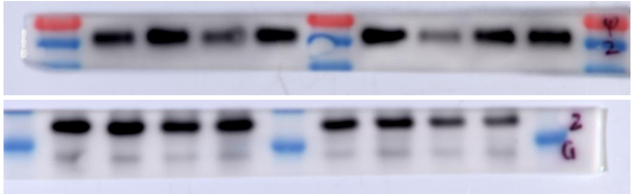

Figure 5E

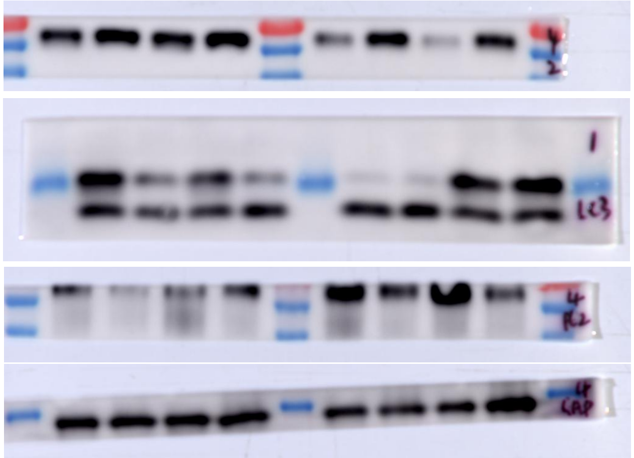

Figure 5F

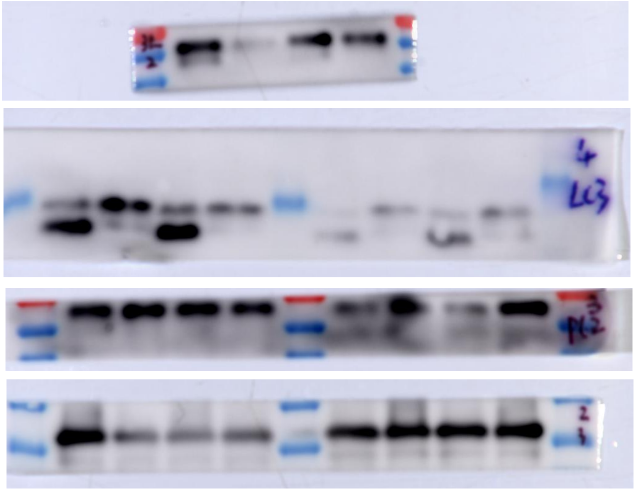

Figure 5I

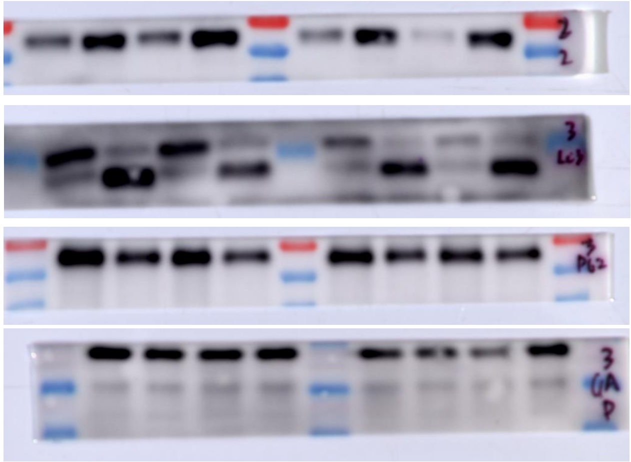

Figure 5J

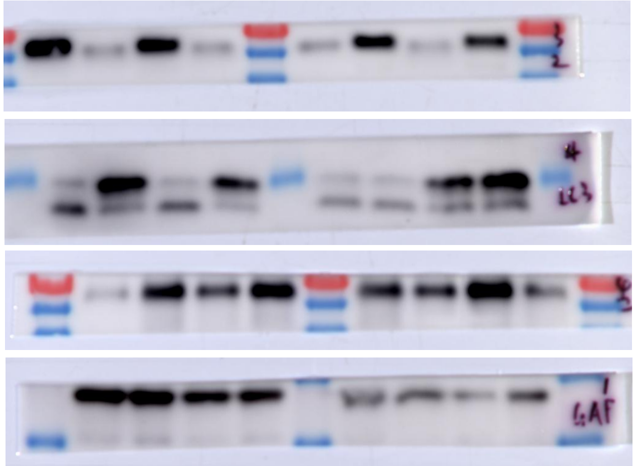

Figure 6E

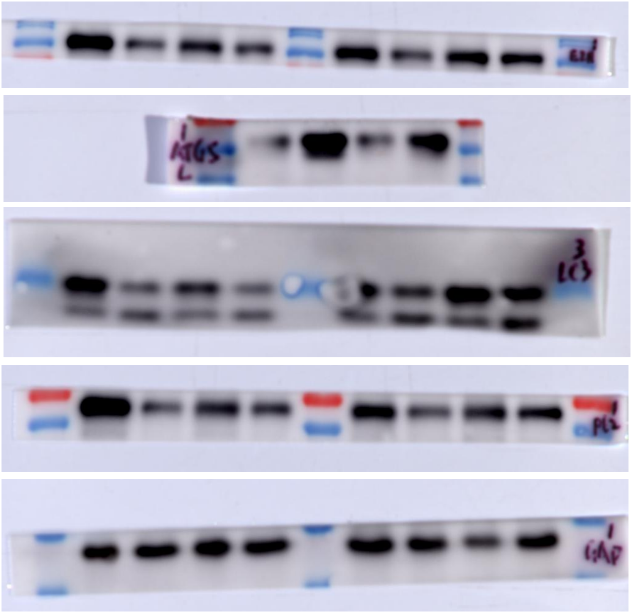

Figure 6F

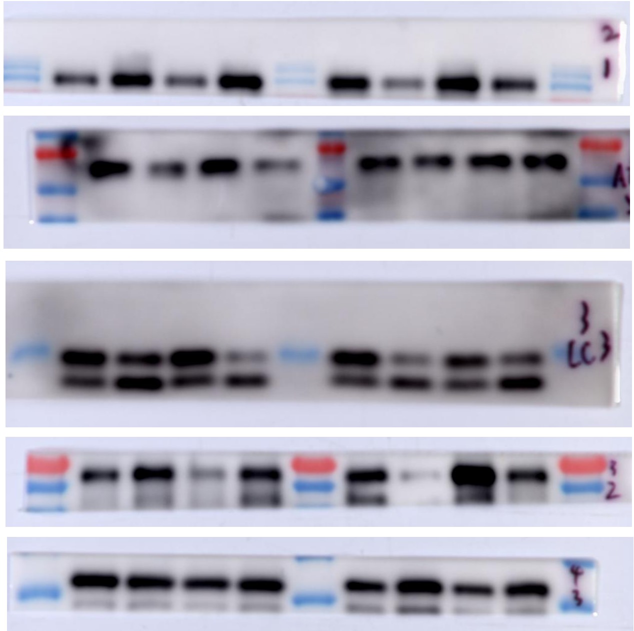

Supplement: Supplementary file 1 [file DataSheet2.PDF]
