# Supplementary material for: LINC-PINT suppresses cisplatin resistance in gastric cancer by inhibiting autophagy activation via epigenetic silencing of ATG5 by EZH2
Source: Front Pharmacol. 2022 Aug 25;13:968223. doi: 10.3389/fphar.2022.968223 (PMC9452659; doi:10.3389/fphar.2022.968223)
Supplement: Supplementary file 2 [file DataSheet3.PDF]

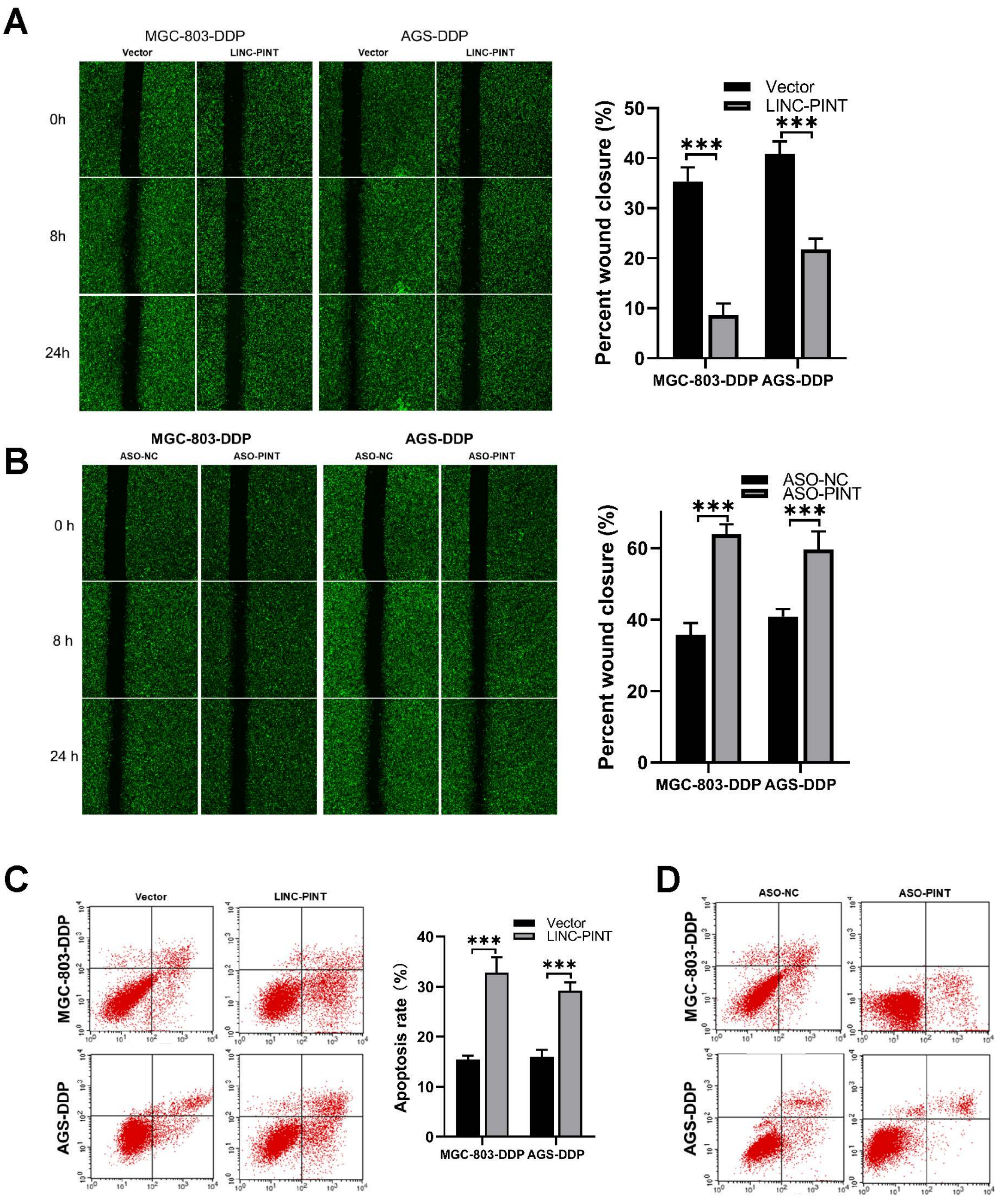

Supplementary Figure 1.

(A) Wound healing assays evaluating the migration ability of LINC-PINT-overexpressing AGS-DDP and MGC-803-DDP cells. (B) Wound healing assays evaluating the migration ability of LINC-PINT-silenced AGS-DDP and MGC-803-DDP cells. (C) Flow cytometry evaluating the apoptosis of LINC-PINT-overexpressing AGS-DDP and MGC-803-DDP cells. (D) Flow cytometry evaluating the apoptosis of LINC-PINT-silenced AGS-DDP and MGC-803-DDP cells. Data were represented as the mean ± SD. *P< 0.05. **P <0.005. ***P <0.001.

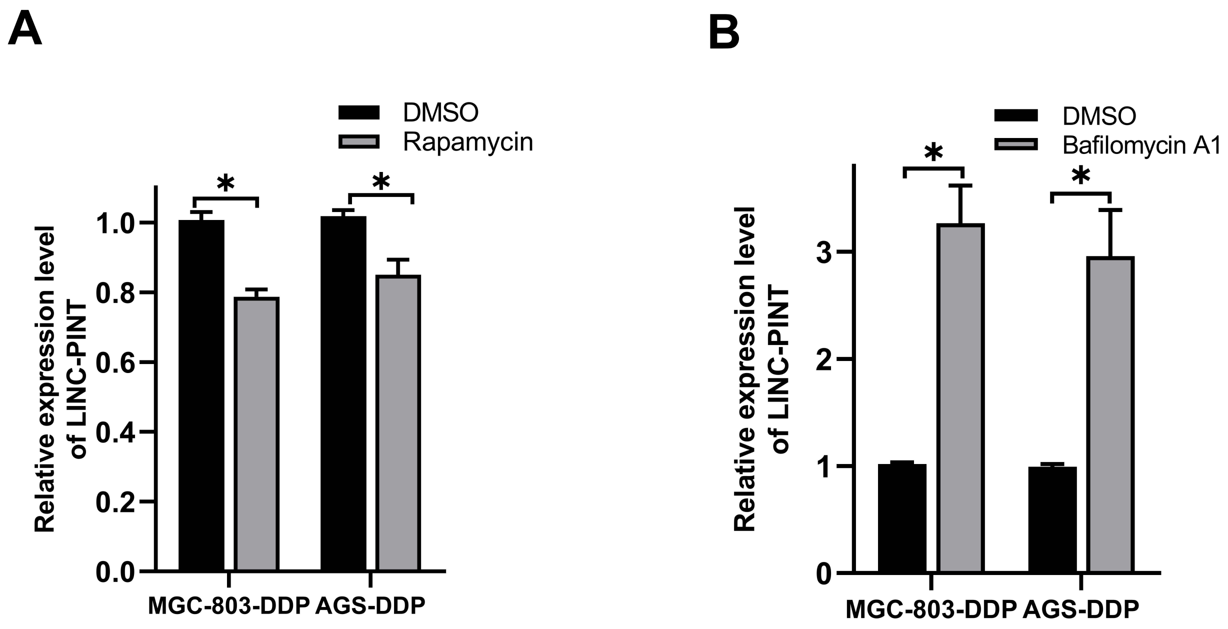

Supplementary Figure 2.

(A) LINC-PINT expression in MGC-803-DDP and AGS-DDP cells after treated with rapamycin. (B) LINC-PINT expression in MGC-803-DDP and AGS-DDP cells after treated with bafilomycin A1. Data were represented as the mean ± SD. *P＜0.05. **P <0.005. ***P <0.001.

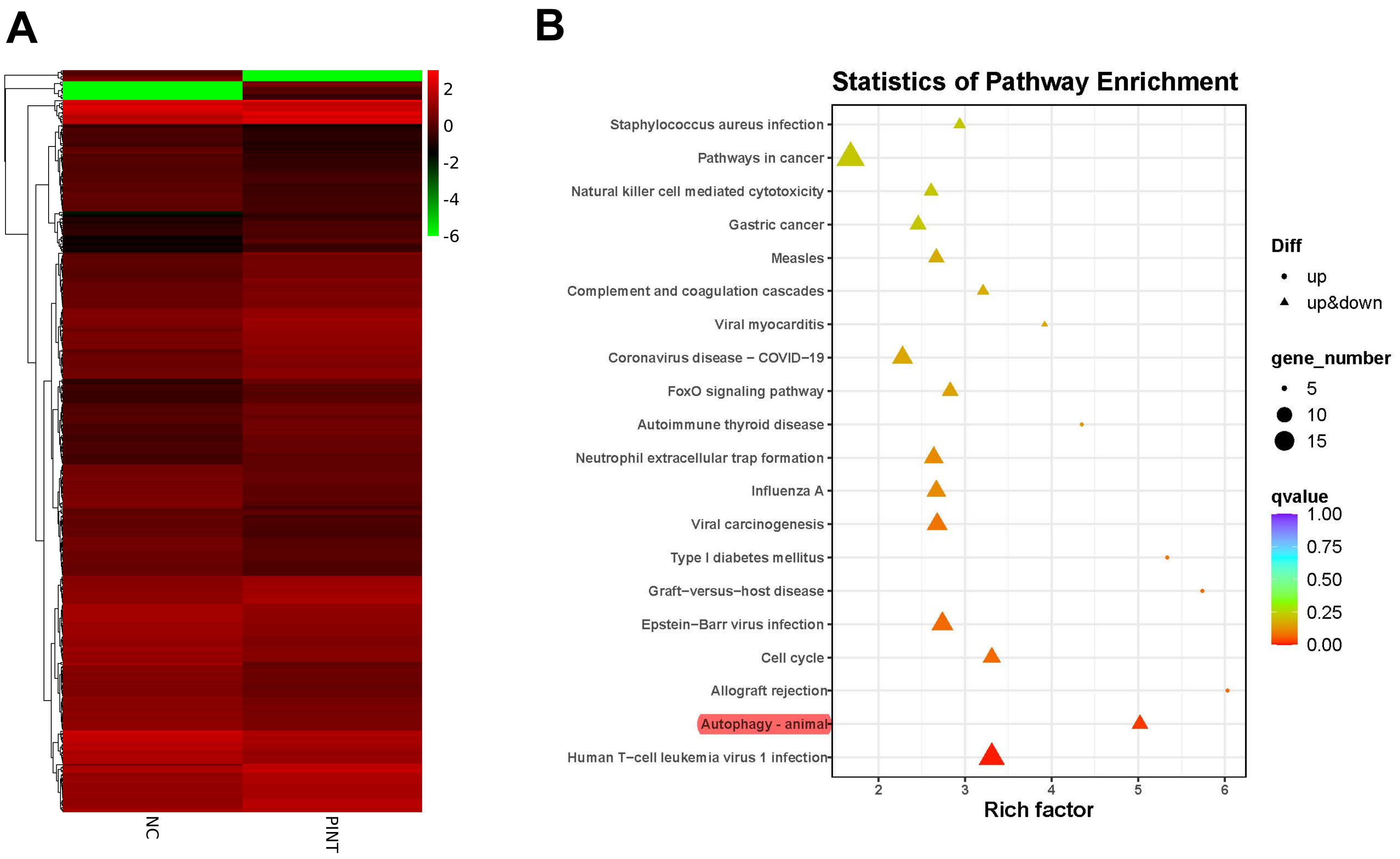

Supplementary Figure 3.
(A) Cluster analysis of differentially expressed genes in LINC-PINT overexpressed DDP-resistant GC cells. (B) KEGG analysis identified multiple pathways in LINC-PINT overexpressed DDP-resistant GC cells.
