# Supplementary material for: LINC-PINT suppresses cisplatin resistance in gastric cancer by inhibiting autophagy activation via epigenetic silencing of ATG5 by EZH2
Source: Front Pharmacol. 2022 Aug 25;13:968223. doi: 10.3389/fphar.2022.968223 (PMC9452659; doi:10.3389/fphar.2022.968223)
Supplement: Supplementary file 3 [file DataSheet1.PDF]

Table S1. Primers sequences

Primer	Sense (5′–3′)	Antisense (5′–3′)
LINC-PINT	CGGTGTAGTGTTCAGCCTCA	GGTGGCAGACTCCTGTTAGC
ATG5	AATCAGGTTTGGTGGAGGCA	CAGTGGAGGAAAGCAGAGGTG
GAPDH	GCACCGTCAAGGCTGAGAAC	TGGTGAAGACGCCAGTGGA
U6	CTCGCTTCGGCAGCACA	AACGCTTCACGAATTTGCGT
β-actin	CTCCATCCTGGCCTCGCTGT	GCTGTCACCTTCACCGTTCC

Table S2. siRNA sequences

EZH2	CCGGCGGCTCCTCTAACCATGTTTACT	AATTCAAAAACGGCTCCTCTAACCATG
